# Genito-Urinary Clinic—Atlanta Medical College, 11 A. M. Thursday

**Published:** 1913-12

**Authors:** A. L. Fowler, J. Calvin Weaver, T. B. Armstrong

**Affiliations:** Associate; Associate; Anaesthetist


### GENITO-URINARY CLINIC—ATLANTA MEDICAL COLLEGE, 11 A. M. THURSDAY.

By Prof. A. L. Fowler; Dr. J. Calvin Weaver, Associate; Dr. T. B. Armstrong, Anaesthetist.

At this clinic was demonstrated the practical use of spinal anaesthesia in surgical diseases of the urinary tract. The anaesthetic employed was tropacocaine.

In a brief clinical lecture it was pointed out that spinal anaesthesia was exhibited to illustrate its practical use in septic cases of the uro-genital tract. It was also mentioned the service rendered by this procedure in patients afflicted with diabetes.

A patient was brought into the clinical amphitheatre suffering from tight stricture of the membranous urethra, whose urine contained pus corpuscles and whose temperature was 101 degrees F. The urine had albumin in a fair amount, an occasional epithelial but more granular casts. The patient passed a small quantity of urine from time to time with the greatest difficulty. There had been a slight hiccough the two days previous.

The patient was requested to sit upon the operating table with his legs hanging on the far side so that the spectators could get a good view of the lumbar region. This was sterilized with water, alcohol and iodine, after which a 3% solution of cocaine was introduced just under the skin over the third lumbar vertebrae. Subsequently a canulla and trocar was passed into the spinal canal at this site and upon the withdrawal of the mandarin spinal fluid issued from the needle. At this juncture a syringe containing 15 minims of tropococaine, made up in adrenalin chloride 1 to 2,000, was attached to the needle and its contents slowly and carefully injected into the spinal canal.

Ten minutes after the introduction of the tropococaine anesthesia was complte, when the operator proceeded to perform an external urethrotomy. This was accomplished in fifteen or twenty minutes, the patient experiencing absolutely not the slightest pain. An internal urethrotomy was also done in the anterior urethra at the same time because of a stricture at the peno-scrotal junction. A drainage tube of large size was introduced into the bladder through the perineal wound and stitched in place. The directions given for the patient as he was about to be sent back to the ward were: "Continue his urinary antiseptic, also his bland fluid diet, and let him have an an abudance of good spring water. He may be nourished now, or after he is placed in bed. The bladder should be irrigated with a pretty warm oxycyanide of mercury solution 1 to 5 or 7,000, about twice daily." Instrumentation in connection with the after treatment was also discussed and the cardinal points stressed upon.

The operator, Dr. Fowler, stated that he had become impressed wit hthe great value of this particular kind of anesthesia, first in Paris, then in Vienna, and lately as a results of his observations while at Doctor Cabot's urological clinic, in Boston during April. He stated that Dr. Cabot employed it often and thought very well of it.

Doctor Fowler, continuing, also said that he thought its greatest value to the urologist was in cases of sepsis of the urinary tract when operative measures for the relief of the condition become necessary. He pointed out its advantages in the prevention of the reflex anuria associated with instrumentation and operation upon the urinary tract. Up to the present time he had employed it in the following operations:

External Urethrotomy, Internal Urethrotomy, Varicocele, Cystoscopy and double catheterization of ureters, Suprapubic Cystotomy, Prostatectomy.

In two recent cases of prostatectomy, in patients 74 or 78 years old, he doubted very seriously if the patients would have stood the operation successfully but for the use of spinal anesthesia.

In the past dozen cases he had noticed only one in which there was the slightest untoward symptoms and this was manifested in nausea and headache several hours after the operation, and was controlled by the exhibition of a small dose of morphine. The case was a varicocele.

Dr. Fowler did not believe that spinal anesthesia should supplant other forms of anesthesia, but he had exhibited it simply to demonstrate that it did have a place in surgery—particularly the surgery of the urinary tract below the kidney.

The other case operated on, in which spinal anesthesia was employed, was a varicocele.

At 2 P. M. Thursday, Dr. John G. Earnest gave a gynecological clinic at Grady Hospital, at which he performed a salpingo oophorectomy. The operation—covering the stumps and closing the abdomen with three rows of stitches was finished in twenty-four minutes.

Dr. L. Sage Hardin, Surgical Clinic, St. Joseph's Infirmary at 2 P. M. Thursday.

Dr. L. C. Fisher, Surgical Clinic at Davis-Fisher Sanatorium, at 2 P. M. Thursday.

Dr. W. B. Emery, gave a Genito-Urinary Clinic, assisted by Drs. E. P. Merritt and J. R. Barfield. Wesley Memorial Hospital, Thursday, Nov. th, 1913.

Case 1.—Edematous Verumontanum with very exaggerated neurotic symptoms. Precocious ejaculations, etc., By the use of the urethroscope the verumontanum was cauterized with a silver nitrate crayon.

Case 2.—Papi loma of the bladder diagnosed and demonstrated with cystoscope.

Case 3.—Perineal urinary fistula due to gonorrhoeal abscess, injected with 50% iodine and glycerine.

Case 4.—Exhibition of ventral hernia following suprapubic prostatectomy.

Case 5.—Hydrocele. Demonstration of subcutaneous injection of serum obtained from the tumor as a curative measure.

Case 6.—Exhibition of case of locomotor ataxia with atony of the bladder.

Case 7.—Demonstration of method of dressing postoperative cases of varicocele with curtailment of scrotum.

The dressing of cases and the administration of Salvarsan to two patients and neosalvarsan to two others completed the clinic.

